# Comparative distribution of extended-spectrum beta-lactamase–producing *Escherichia coli* from urine infections and environmental waters

**DOI:** 10.1371/journal.pone.0224861

**Published:** 2019-11-07

**Authors:** Anna Fagerström, Paula Mölling, Faisal Ahmad Khan, Martin Sundqvist, Jana Jass, Bo Söderquist

**Affiliations:** 1 Department of Laboratory Medicine, Faculty of Medicine and Health, Örebro University, Örebro, Sweden; 2 The Life Science Centre–Biology, School of Science and Technology, Örebro University, Örebro, Sweden; Amphia Ziekenhuis, NETHERLANDS

## Abstract

Extended-spectrum beta-lactamase (ESBL)–producing *Escherichia coli* have been reported in natural environments, and may be released through wastewater. In this study, the genetic relationship between ESBL-producing *E*. *coli* collected from patient urine samples (n = 45, both hospitalized patients and out-patients) and from environmental water (n = 82, from five locations), during the same time period, was investigated. Three independent water samples were collected from the municipal wastewater treatment plant, both incoming water and treated effluent water; the receiving river and lake; and a bird sanctuary near the lake, on two different occasions. The water was filtered and cultured on selective chromID ESBL agar plates in order to detect and isolate ESBL-producing *E*. *coli*. Illumina whole genome sequencing was performed on all bacterial isolates (n = 127). Phylogenetic group B2 was more common among the clinical isolates than the environmental isolates (44.4% vs. 17.1%, *p* < 0.01) due to a significantly higher prevalence of sequence type (ST) 131 (33.3% vs. 13.4%, *p* < 0.01). ST131 was, however, one of the most prevalent STs among the environmental isolates. There was no significant difference in diversity between the clinical isolates (DI 0.872 (0.790–0.953)) and the environmental isolates (DI 0.947 (0.920–0.969)). The distribution of ESBL genes was similar: *bla*_CTX-M-15_ dominated, followed by *bla*_CTX-M-14_ and *bla*_CTX-M-27_ in both the clinical (60.0%, 8.9%, and 6.7%) and the environmental isolates (62.2%, 12.2%, and 8.5%). Core genome multi-locus sequence typing showed that five environmental isolates, from incoming wastewater, treated wastewater, Svartån river and Hjälmaren lake, were indistinguishable or closely related (≤10 allele differences) to clinical isolates. Isolates of ST131, serotype O25:H4 and fimtype *H30*, from the environment were as closely related to the clinical isolates as the isolates from different patients were. This study confirms that ESBL-producing *E*. *coli* are common in the aquatic environment even in low-endemic regions and suggests that wastewater discharge is an important route for the release of ESBL-producing *E*. *coli* into the aquatic environment.

## Introduction

The increasing prevalence of extended-spectrum beta-lactamase (ESBL)–producing *Enterobacterales* is a serious problem worldwide. ESBL-producing bacteria are resistant to most beta-lactam antibiotics, including cephalosporins, and they are often co-resistant to other antibiotics such as fluoroquinolones, aminoglycosides, and trimethoprim [1]. ESBL-producing *Escherichia coli* are found globally in both hospital and community settings as a cause of urinary tract infections and bloodstream infections [2]. During the last decade, ESBL-producing *E*. *coli* have been isolated from wastewater, surface water from rivers and lakes, and other recreational waters [3–7]. These bacteria have also been found in wild animals, livestock and companion animals, which have been suggested to contribute to the spread of ESBL-producing bacteria in the environment [8–14]. Recent studies have shown that approximately 4%–7% of the Swedish population are asymptomatic carriers of ESBL-producing *E*. *coli*, indicating that humans are an important source of ESBL-producing *E*. *coli* contamination of the environment [15, 16]. Importantly, the environmental pollution of ESBL-producing *E*. *coli* from animal and human waste is not only an issue in developing countries with poor sanitation, as these bacteria can also be released into the environment through treated effluent water from wastewater treatment plants (WWTP) [4, 17].

The global spread of ESBL-producing *E*. *coli* has been dominated by the highly successful *E*. *coli* lineage with sequence type 131 (ST131). This lineage is commonly found not only in clinical isolates from humans but also in animals and the environment [5, 18, 19]. Different sub-lineages are found within ST131, characterized by genetic variations in the type 1 fimbrial adhesion gene *fimH*, of which *fimH*30 is the most prevalent and appears to be the most important lineage involved in the pandemic spread of the ESBL gene *bla*_CTX-M-15_ [18]. ESBLs of the CTX-M group are the dominant ESBL enzymes globally, and CTX-M-15 is the most common in *E*. *coli* causing human infections [20]. In Örebro County, located in central Sweden, 27% of all clinical CTX-M–producing *E*. *coli* isolated between 1999 and 2008 belonged to ST131, and the majority of these carried *bla*_CTX-M-15_ [21]. In samples from water used as source material for drinking water supply, collected in 2012 from the same region as the present study, 1%–4% of the total amount of *E*. *coli* was producing ESBL or transferrable AmpC beta-lactamases, but none of these were typed to ST131 [22].

Although ESBL-producing *E*. *coli* has been reported in environmental water in both high- and low-prevalence countries, there are only a few studies that have investigated the relationship between human and environmental isolates with a spatial and temporal association [7, 23]. Therefore, the aim of the present study was to investigate the genetic relationship between ESBL-producing *E*. *coli* isolated from humans and the aquatic environment during the same time period in a low-endemic setting, using whole genome sequencing.

## Material and methods

### Study setting

The study was performed in Örebro, a mid-sized city located in central Sweden with a population of approximately 153,000 inhabitants (www.scb.se/en, accessed on 22 Feb-2019). The municipal WWTP processes a total of 45,000 m^3^ of wastewater per day (www.orebro.se), from households, one university hospital, local industries, and a small airport. After treatment, effluent from the WWTP is discharged into Svartån River, which flows through the urban area and terminates in Hjälmaren Lake. The majority of the agricultural activities are outside of the urban area, and there are no pharmaceutical industries in Örebro city. There are 16 primary care centers located in the city and one large tertiary hospital (Örebro University Hospital). The hospital wastewater is transported to the municipal WWTP without prior treatment.

### Water sample collection and isolation of ESBL-producing *E*. *coli*

**Fig 1:**
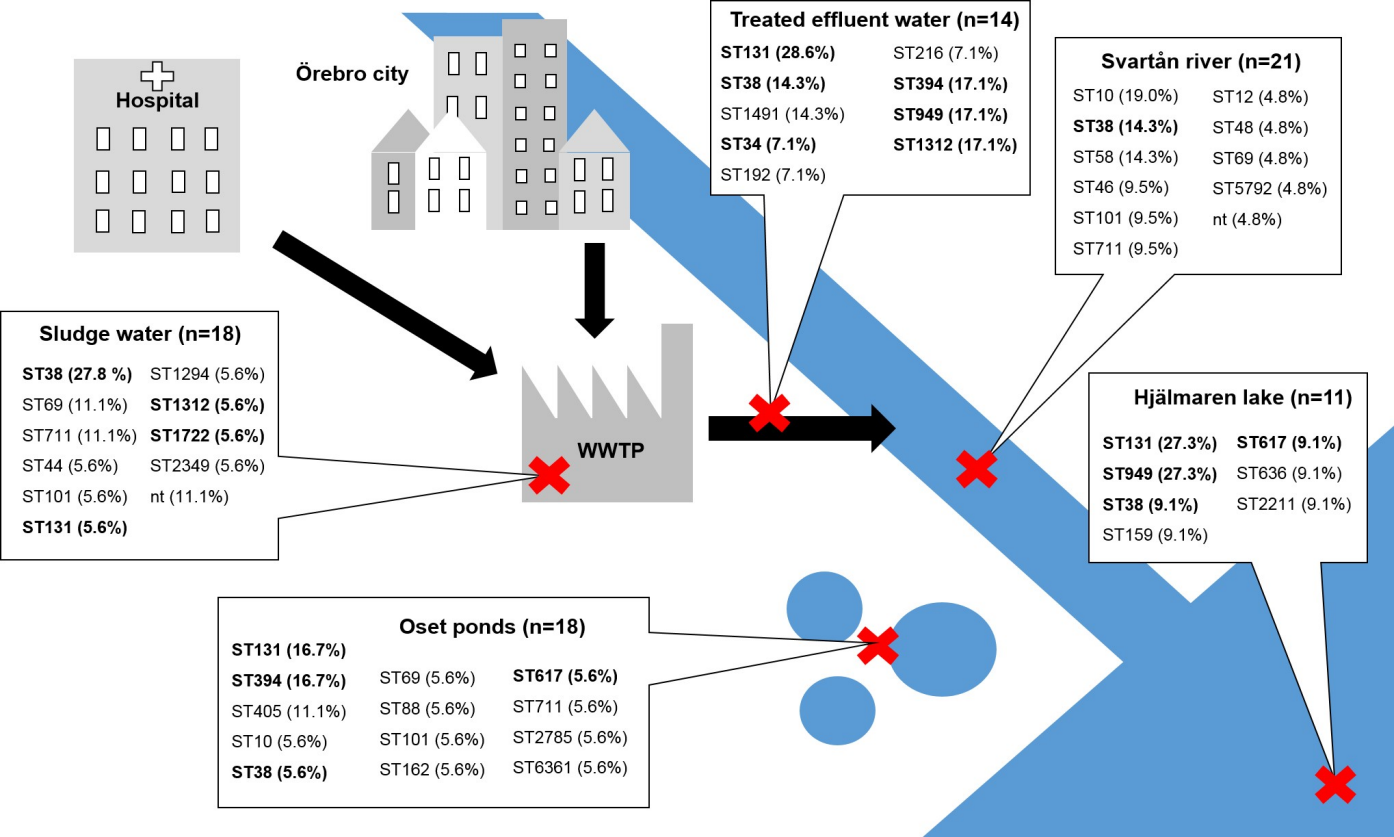
Schematic map of the study area. The red crosses indicate the sampling locations at the wastewater treatment plant (WWTP) and in Svartån River, Hjälmaren Lake, and Oset ponds. Six water samples were collected from each sampling site (three in June and three in October). Presented in the figure are also the number of isolates included from each site and the sequence types (ST) found in the respective locations., STs in bold were also found among the clinical isolates.

The water samples (n = 30) were collected on one occasion in June and one occasion in October 2013. Wastewater samples, from the incoming wastewater to the WWTP after the mechanical removal of solid waste, and from effluent water after treatment, were collected at three separate times over a 12 h period, at each sampling occasion. The environmental water samples were collected from Svartån River, downstream of the wastewater effluent discharge (59°16'42.5"N 15°15'41.5"E); from Hjälmaren Lake, approximately 1 nautical mile downstream of the wastewater effluent discharge (59°16'40.2"N 15°17'31.1"E); and from several ponds in Oset, a migratory bird sanctuary near, but not connected to, the lake (Fig 1). No permit or permission was required for the sampling of waters according to “the everyman’s right” (allemansrätten) in the Swedish constitution. For environmental waters, at each sampling occasion, three independent surface-water samples (approx. 50 cm below the surface) of 1 L from each site were collected across the flow of the water, in sterile borosilicate glass bottles and stored at 6°C prior to processing within 24 hours.

ESBL-producing *E*. *coli* were isolated from environmental waters by filtering 100 ml of 1, 1/10, and 1/100 dilution of each sample through 0.45 μm polyethylene sulfonate membrane filters (Sartorius Stedim Biotech, Sweden) and placed onto selective chromID ESBL agar plates (bioMérieux, Marcy-l’Etoile, France). The agar plates were incubated for 18 h at 37°C. Presumptive *E*. *coli* colonies, (pink colonies on chromID ESBL agar), were sub-cultured onto Chromocult Coliform Agar (Merck, Darmstadt, Germany) to confirm the species and to obtain pure isolates. Isolates identified as *E*. *coli* on the Chromocult agar (dark blue to violet colonies) were stored at -80°C until further analysis. From the sampling in June, all colonies identified as *E*. *coli* from each sample were included for further analysis (1–12 per sampling site), while for the October sampling, all isolates identified as *E*. *coli* were included, except for samples with >10 isolates, where ten isolates were picked randomly for further analysis. Species ID was verified with matrix-assisted laser desorption/ionization time-of-flight mass spectrometry (MALDI-TOF MS) using Microflex LT and Biotyper 3.1 (Bruker Daltonics, Bremen, Germany), according to the manufacturer’s instructions. The production of ESBL was verified using double-disk synergy testing on Mueller-Hinton II agar 3.8 w/v (BD Diagnostic Systems, Sparks, MD, USA), as previously described [24]. The ESBL-producing *E*. *coli* isolates (n = 82) were stored at -80°C until further analysis.

### Isolation of ESBL-producing *E*. *coli* from clinical samples

*E*. *coli* with phenotypic ESBL production from routine urine samples sent to the Department of Laboratory Medicine, Clinical Microbiology, University Hospital, Örebro, during February to September 2013, were identified and isolated with routine diagnostic methods. The urine samples came from both hospitalized patients and outpatients. Isolates from all patients infected with ESBL-producing *E*. *coli* for the first time were included in the study (n = 45). Bacterial species identification was performed with MALDI-TOF MS (Bruker Daltonics), and *E*. *coli* isolates were investigated for cephalosporin resistance using disk diffusion according to EUCAST methodology (http://www.eucast.org). Verification of ESBL production and storage was performed as described for the environmental isolates.

### Whole genome sequencing

Bacterial DNA was extracted using the UltraClean microbial DNA isolation kit (MO BIO Laboratories, Carlsbad, CA, USA) according to the manufacturer’s instructions. DNA concentrations were measured using a Qubit fluorometer (Life Technologies, Waltham, MA, USA) and the Qubit dsDNA BR and the Qubit dsDNA HS assay kits (Life Technologies). A DNA library was prepared using Nextera XT DNA library preparation kit with the Nextera XT index kit (Illumina, San Diego, CA, USA) according to the manufacturer’s instructions. Library fragment lengths were assessed on a 2100 Bioanalyzer using the high sensitivity DNA kit (Agilent Technologies, Santa Clara, CA, USA), and the library was subsequently sequenced on a MiSeq sequencer (Illumina) using the MiSeq reagent kit v3, 600-cycle (Illumina). Quality trimming of reads was performed using the FastQ toolkit v 2.0.0 on the Illumina BaseSpace server (BaseSpace Labs) with a Q threshold set to ≥20. *De novo* genome assembly of paired-end reads was performed using SPAdes Genome Assembler v 3.6.0 default settings [25]. A minimum coverage >30-fold and N50 >50,000 was considered acceptable.

Raw sequencing reads was deposited in the NCBI Sequence Read Archive (SRA) under BioProject accession number PRJNA558173.

### Whole genome data analysis

Phylogenetic groups were determined by *in silico* PCR using primer sequences described by Clermont et al. [26].

Ridom SeqSphere+ 3.4.0 (Ridom GmbH, Münster, Germany) software was used to determine the multi-locus sequence type (MLST) (Warwick) and to create a local ad hoc core genome MLST (cgMLST) scheme. The fully sequenced genome of *E*. *coli* CFT073 (NC_004431.1) was used as a reference genome for the cgMLST scheme. Open reading frames (ORFS) were extracted from the reference genome, and seven additional *E*. *coli* query genomes (NC_020163.1, NZ_CP009644.1, NC_009800.1, NC_022648.1, NZ_CP016007.1, NZ_CP015834.1, NZ_CP009578.1), using the cgMLST Target Definer v 1.4 in SeqSphere+. Default settings were used to extract genes that were non-homologous and non-overlapping, had a valid start/stop codon in the reference genome, appeared uniquely in all query genomes (sequence identity >90%, alignment 100%), and did not have invalid stop codons in >80% of the query genomes. An excluded sequences filter was applied to discard plasmid sequences. Out of the resulting 2758 genes, 1957 genes were found in all isolates included in this study except for one, and were therefore defined as the core genome. One environmental isolate (SN1013-18) that contained <95% of the core genes was excluded from the cgMLST analysis. A separate cgMLST analysis was performed on isolates that belonged to ST131. Out of the 2758 genes in the cgMLST scheme, 2682 genes were identified in all ST131 isolates and included in the analysis. Neighbor-joining (NJ) trees and Minimum spanning trees were constructed based on a distance matrix that was built from pairwise allelic profile comparisons of the core genes in all isolates included in the analysis. Discriminatory index (Simpson’s index of diversity) (DI (95% CI)) was calculated based on MLST classification in Ridom SeqSphere+ [27–29].

For ESBL genotyping and screening for other acquired antibiotic resistance genes, ResFinder v 2.1 server was used (Center for Genomic Epidemiology, Technical University of Denmark, Lyngby, Denmark) with the following settings: selected ID threshold 98.0%, selected minimum length 60% [30]. All ST131 and other closely related isolates (≤10 cgMLST allele differences), were also analyzed using VirulenceFinder 1.5 and PlasmidFinder 1.3, with default settings [31, 32]. The FimTyper 1.0 and SerotypeFinder 1.1 (Center for Genomic Epidemiology) were used with default settings to determine *fimH* type and serotype in isolates with sequence type ST131 [33].

To statistically compare the clinical and the environmental groups, Chi-square tests were performed, and the significance level was set to *p <* 0.05.

### Ethics

This study used bacterial isolates from humans. No tissue material or other biological material was stored from the patients. All information regarding these isolates was anonymized.

## Results

ESBL-producing *E*. *coli* were found in 100% of water samples (30/30) collected in the aquatic environment in Örebro city. A total of 82 environmental isolates were included from the WWTP (n = 32, from 12 samples) and environmental waters (n = 50, from 18 samples), and 45 clinical isolates (from 45 patients) from both hospitalized patients and out-patients. All 127 isolates were submitted for whole genome sequencing. The phylogenetic analysis showed that all four phylogenetic groups (A, B1, B2, and D) were represented in both the clinical and the environmental isolates, but group B2 was significantly more common among the clinical isolates (*p* < 0.01), Table 1. Based on the MLST analysis, there was no significant difference in diversity, as 34 different STs were found among the 82 environmental isolates (DI 0.947 (0.924–0.969)), whereas the corresponding values for the clinical isolates were 21 STs from a total of 45 isolates (DI 0.872 (0.790–0.953)).

**Table 1:** Distribution of phylogenetic group, sequence type (ST), and ESBL genotype in clinical isolates and isolates from water environments.

	Clinical isolates			Environmental isolates			*p-value* Clinical vs environmental isolates			*p-value* Environmental isolates
	Total *n* = 45, (%)	Feb–May *n* = 26, (%)	June–Sept *n* = 19, (%)	Total *n* = 82, (%)	June *n* = 36, (%)	October *n* = 46, (%)	Total vs total	Feb–May vs June	June–Sept vs October	June vs October
**Phylogenetic group**										
A	6 (13.3)	4 (15.4)	2 (10.5)	23 (28.0)	2 (5.6)	21 (45.7)	ns	ns	<0.01	<0.01
B1	5 (11.1)	2 (7.7)	3 (15.8)	20 (24.4)	16 (44.4)	4 (8.7)	ns	<0.01	ns	<0.01
B2	20 (44.4)	14 (53.8)	6 (31.6)	14 (17.1)	4 (11.1)	10 (21.7)	<0.01	<0.01	ns	ns
D	14 (31.1)	6 (23.1)	8 (42.1)	25 (30.5)	14 (38.9)	11 (23.9)	ns	ns	ns	ns
**Sequence type**										
ST131	15 (33.3)	11 (42.3)	4 (21.1)	11 (13.4)	3 (8.3)	8 (17.4)	<0.01	<0.01	ns	ns
ST38	6 (13.3)	2 (7.7)	4 (21.1)	12 (14.6)	5 (13.9)	7 (15.2)	ns	ns	ns	ns
Other	24 (53.3)	13 (50.0)	11 (57.8)	59 (72.0)	28 (77.8)	31 (67.4)	<0.05	<0.05	ns	ns
**ESBL genotype**										
CTX-M-15	28 (62.2)	19 (73.1)	9 (47.4)	51 (62.2)	20 (55.6)	31 (67.4)	ns	ns	ns	ns
CTX-M-14	3(6.7)	2 (7.7)	1 (5.3)	10 (12.2)	4 (11.1)	6 (13.0)	ns	ns	ns	ns
CTX-M-27	3 (6.7)	1 (3.8)	2 (10.5)	7 (8.5)	3 (8.3)	4 (8.7)	ns	ns	ns	ns
Other	11 (24.4)	4 (15.4)	7 (36.8)	14 (17.1)	9 (25.0)	5 (10.9)	ns	ns	<0.05	ns

ns = not significant (*p* > 0.05)

Eleven ESBL genotypes were detected in all locations. The most common was *bla*_CTX-M-15_ in both the clinical and the environmental isolates, followed by *bla*_CTX-M-14_ and *bla*_CTX-M-27_, Table 1. The remaining types were found only sporadically. No acquired genes encoding carbapenemases or resistance to colistin were found.

**Fig 2:**
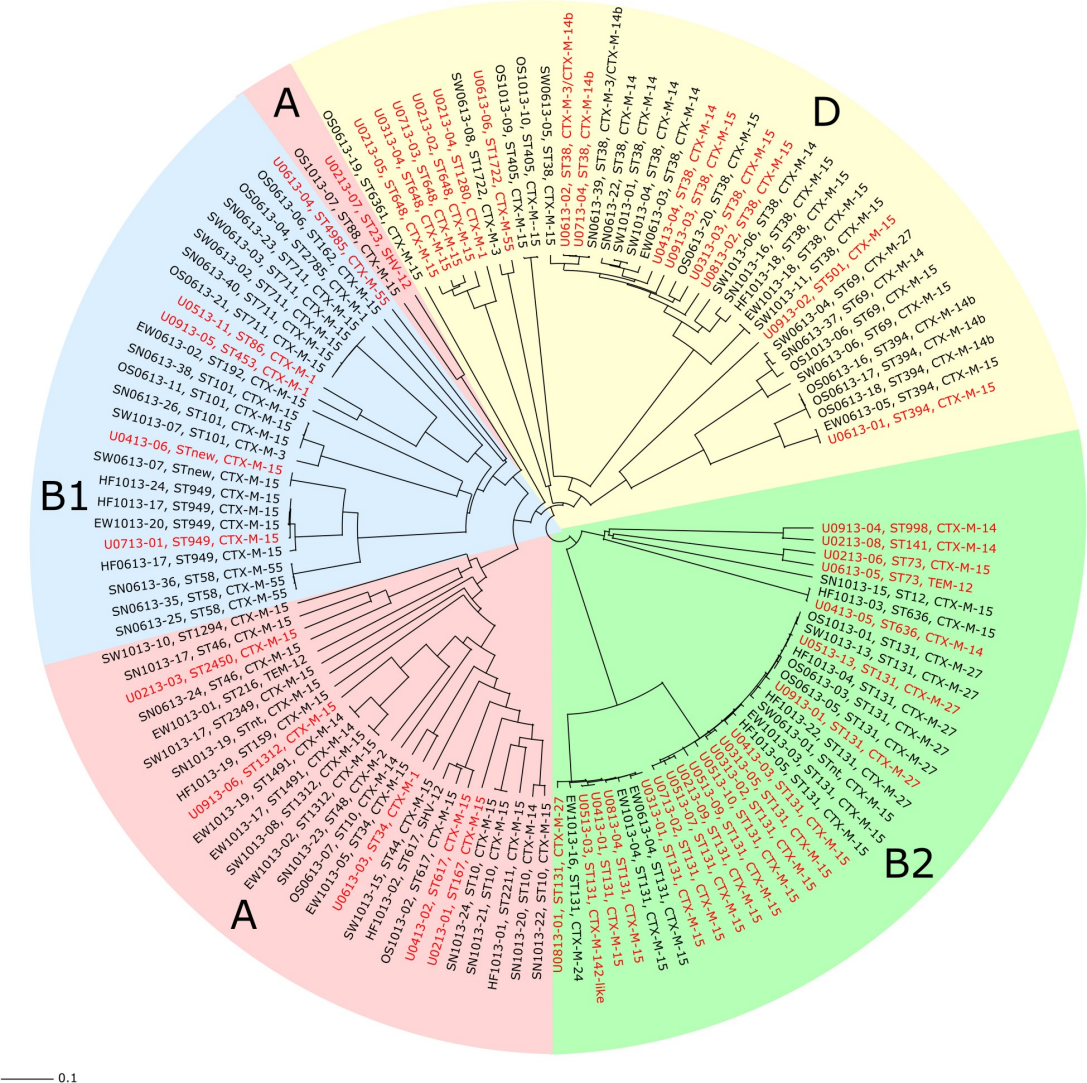
Neighbor–joining (NJ) tree based on core genome multi–locus sequence typing (cgMLST) analysis. The NJ tree is based on allele differences in 1957 core genes found in 126 ESBL–producing *E*. *coli* isolates included in the cgMLST analysis. Isolate ID, ST, and ESBL type are written in red for the clinical isolates (n = 45), and in black for the environmental isolates (n = 81). The colored areas represent the different phylogenetic groups.

**Fig 3:**
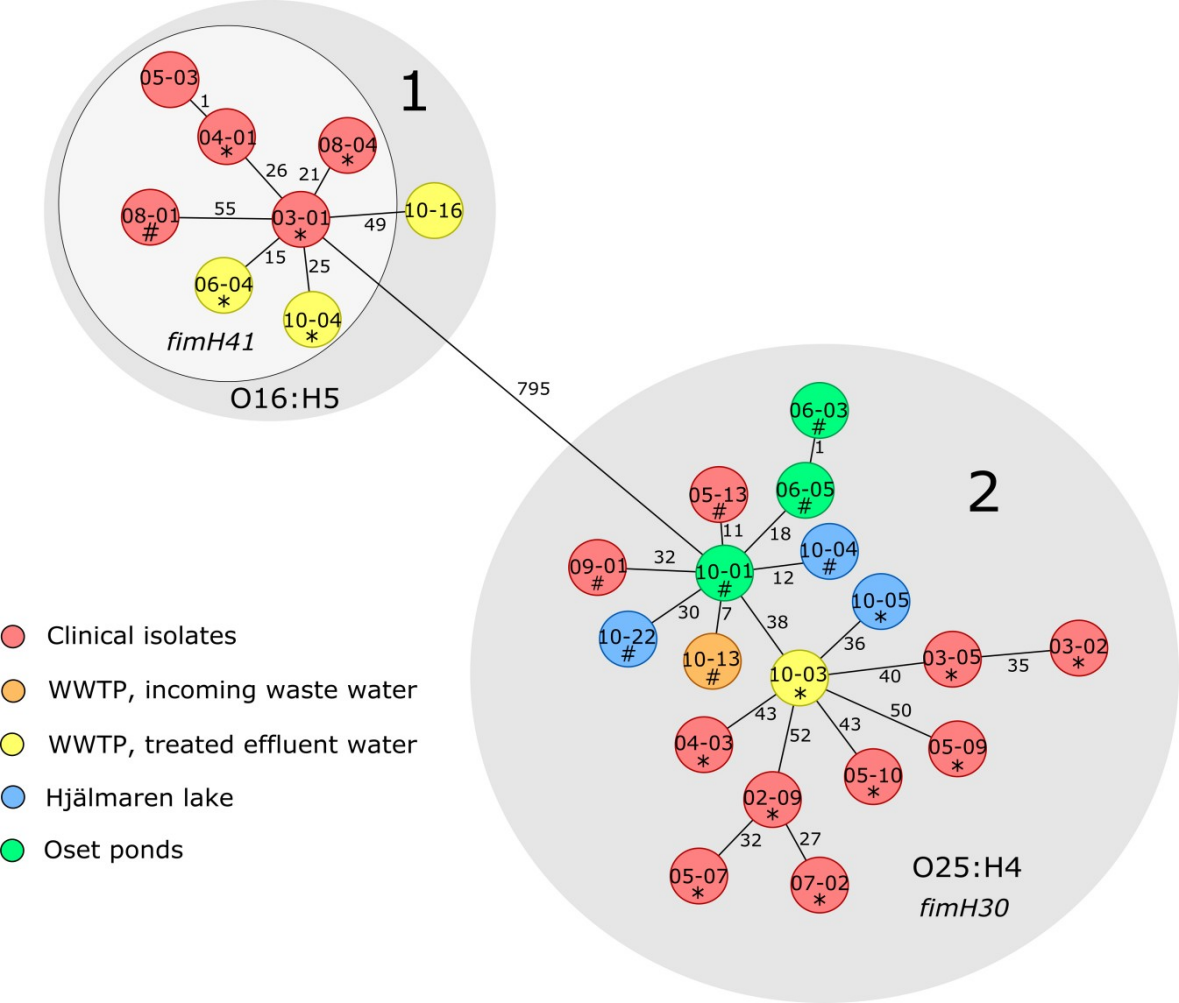
Minimum spanning tree (MST) of the ST131 isolates. The MST shows all ST131 isolates found in patients and the environment (n = 26) and is based on allele differences in 2682 core genes. Each circle represents one isolate. Isolates marked with * carried *bla*_CTX–M–15_ and isolates marked with # carried *bla*_CTX–M–27._ The different colors show the sampling locations and the numbers next to the lines connecting the circles show the number of allele differences between the isolates.

To determine the genetic relationship between the clinical isolates and those from the various environments, cgMLST was conducted. A neighbor-joining tree was constructed based on the cgMLST results (Fig 2). Clinical and environmental isolates were interspersed throughout the tree, and the main branches were almost in complete agreement with the four phylogenetic groups and the individual STs that also clustered together. ST131 and ST38 were the two most prevalent STs found in both urine samples and environmental samples (Table 1). The ST38, which belonged to phylogenetic group D, was found at all sampling sites in the environment, and there was no significant difference in the proportion of ST38 between the clinical and the environmental isolates. As shown in Fig 2, phylogenetic group B2 was dominated by ST131, which was found in 1 of 18 (5.6%) isolates from sewage sludge, 4 out of 14 (28.6%) isolates from treated effluent water, 3 out of 11 (27.3%) isolates from Hjälmaren Lake, and 3 out of 18 (16.7%) isolates from Oset ponds. A significantly higher proportion of ST131 was found among the clinical isolates than among the isolates from the environment (*p* < 0.01) (Table 1). The cgMLST analysis divided ST131 into two sub-lineages (1 and 2), with 795 allele differences in between, Fig 3. *In silico* serotyping and Fim typing revealed that all isolates in lineage 1 were of serotype O16:H5, and all except one carried *fimH*41. The majority (5/8, 62.5%) of the isolates within this lineage were of clinical origin, and all three environmental isolates were obtained from treated effluent water from the WWTP. The isolates found within lineage 2 were *fimH*30 positive and belonged to serotype O25:H4. This lineage contained clinical isolates (10/18, 55.6%) and isolates from all sampling locations in the environment, except for the river. A significantly higher frequency of the ESBL gene *bla*_CTX-M-27_ was found among the environmental ST131-O25:H4 isolates compared to the clinical isolates of this lineage (6/8 vs 2/10, *p <* 0.05). Further analysis of acquired resistance genes, virulence genes and plasmid replicon types in the ST131 isolates showed that one urine isolate (03–01) within lineage 1 had identical resistance gene- and plasmid replicon profiles as an isolate from treated effluent water (06–04), Table 2. Among the isolates that carried *bla*_CTX-M-15_ within lineage 2 there was a large variation in both resistance-, virulence-, and plasmid profiles. The *bla*_CTX-M-27_ positive isolates within lineage 2 were very similar to each other, and one of the urine isolates (05–13) had identical virulence-, resistance-, and plasmid profiles as five of the environmental isolates.

**Table 2:** Comparison of ST131 isolates.

Isolate-ID	Source	Month of isolation	Serotype	*FimH*	Virulence genes	Acquired resistance genes	Plasmid replicon types
**ST131 Lineage 1**							
03–01	Urine	March	O16:H5	41	iss, senB, nfaE, iha, sat	CTX-M-15, TEM-1B, strA, strB, aac(3)-Iid, aadA5, mph(A), sul1, sul2, tet(A), dfrA17	IncFII(29), IncFIA, IncFIB(AP001918), Col156
04–01	Urine	April	O16:H5	41	nfaE, iha, sat	CTX-M-15, TEM-1B	IncFII(29), IncFIB(AP001918)
05–03	Urine	May	O16:H5	41	nfaE, iha, sat	CTX-M-142-like, TEM-1B	IncFII(29), IncFIB(AP001918)
06–04	Effluent water from WWTP	June	O16:H5	41	iss, senB, nfaE, iha	CTX-M-15, TEM-1B, strA, strB, aac(3)-Iid, aadA5, mph(A), sul1, sul2, tet(A), dfrA17	IncFII(29), IncFIA, IncFIB(AP001918), Col156
08–01	Urine	August	O16:H5	41	senB, iha, sat	CTX-M-27, strA, strB, aadA5, mph(A), sul1, sul2, tet(A), dfrA17	IncFII(29), IncFIB(AP001918), Col156
08–04	Urine	August	O16:H5	41	senB, nfaE, iha	CTX-M-15, TEM-1B, strA, strB, aadA1, sul1, tet(A), dfrA1	IncFII, IncFIA, IncFIB(AP001918), IncFIC(FII), Col156
10–04 (EW)	Effluent water from WWTP	October	O16:H5	41	gad, senB, iha, sat	CTX-M-15, TEM-1B	IncFII(29), IncFIB(AP001918), Col156
10–16	Effluent water from WWTP	October	O16:H5	89	senB, nfaE, iha, astA, sat	CTX-M-24, TEM-1B	IncFII(29), IncFIB(AP001918), Col156
ST131 Lineage 2 with *bla*_CTX-M-15_							
02–09	Urine	February	O25:H4	30	gad, iss, iha, sat, cnf1	OXA-1, aac(3)-IIa, aac(6')Ib-cr, mph(A), catB3, sul1, sul2, tet(A)	IncFII, IncFIA, IncFIB(AP001918)
03–02	Urine	March	O25:H4	30	gad, iss, iroN, cma, mchF	TEM-1B, OXA-1, strA, strB, aac(3)-IIa, aac(6')Ib-cr, catB3, sul2, tet(A), dfrA14	IncFII, IncFIA, IncFIB(AP001918)
03–05	Urine	March	O25:H4	30	gad, iss, iha, sat, iroN, cma, mchF	TEM-1B, OXA-1, strA, strB, aac(3)-IIa, aac(6')Ib-cr, catB3, sul2, tet(A), dfrA14	IncFII, IncFIA, IncFIB(AP001918), IncX1
04–03	Urine	April	O25:H4	30	gad, iss, nfaE, iha, sat	OXA-1, aadA5, aac(6')Ib-cr, mph(A), catB3, sul1, tet(A), dfrA17	IncFII, IncFIA, IncI1, Col(BS512)
05–07	Urine	May	O25:H4	30	gad, iss, iha, sat, cnf1	TEM-1B, OXA-1, aac(3)-Iia, aadA2, aac(6')Ib-cr	IncFII(pRSB107), IncFIB(AP001918), IncHI2, IncHI2A, ColRNAI
05–09	Urine	May	O25:H4	30	gad, iss, senB, iha, astA, sat	TEM-1B, OXA-1, aac(3)-Iia, aac(6')Ib-cr, catB3	IncFII(pRSB107), IncFIA, IncFIB(AP001918), IncI1, Col156
05–10	Urine	May	O25:H4	30	gad, iss, iha, sat	OXA-1, aac(6')Ib-cr	IncFII(pRSB107)
07–02	Urine	July	O25:H4	30	gad, iss, senB, iha, sat, cnf1	OXA-1, aac(3)-Iia, aadA5, aac(6')Ib-cr, mph(A), catB3, sul1, dfrA17	IncFII, IncFIA, IncFIB(AP001918), Col156
10–03	Effluent water from WWTP	October	O25:H4	30	gad, iss, iha, sat	TEM-1B, tet(A)	IncFII(pRSB107), IncFIA
10–05	Hjälmaren Lake	October	O25:H4	30	gad, iss, senB, nfaE, iha, sat	aadA5, mph(A), sul1, dfrA17	IncFIA, IncFIB(AP001918), Col156
ST131 Lineage 2 with *bla*_CTX-M-27_							
05–13	Urine	May	O25:H4	30	gad, iss, senB, iha, sat	strA, strB, aadA5, mph(A), sul1, sul2, tet(A), dfrA17	IncFII(pRSB107), IncFIA, IncFIB(AP001918), Col156
06–03	Oset ponds	June	O25:H4	30	gad, iss, senB, iha, sat	strA, strB, aadA5, mph(A), sul1, sul2, tet(A), dfrA17	IncFII(pRSB107), IncFIA, IncFIB(AP001918), Col156
06–05	Oset ponds	June	O25:H4	30	gad, iss, senB, iha, sat	strA, strB, aadA5, mph(A), sul1, sul2, tet(A), dfrA17	IncFII(pRSB107), IncFIA, IncFIB(AP001918), Col156
09–01	Urine	September	O25:H4	30	gad, iss, senB		IncFII(pRSB107), IncFIA, IncFIB(AP001918), Col156, Col(BS512)
10–13	Sludgewater in WWTP	October	O25:H4	30	gad	strA, strB, aadA5, mph(A), sul1, sul2, tet(A), dfrA17	IncFII(pRSB107), IncFIA, IncFIB(AP001918), Col156
10–04 (HL)	Hjälmaren lake	October	O25:H4	30	gad, iss, senB, iha, sat	strA, strB, aadA5, mph(A), sul1, sul2, tet(A), dfrA17	IncFII(pRSB107), IncFIA, IncFIB (AP001918), Col156, Col(IMGS31)
10–22	Hjälmaren lake	October	O25:H4	30	gad, iss, senB, iha, sat	strA, strB, aadA5, mph(A), sul1, sul2, tet(A), dfrA17	IncFII(pRSB107), IncFIA, IncFIB(AP001918), Col156
10–01	Oset ponds	October	O25:H4	30	gad, iss, senB, iha, sat	strA, strB, aadA5, mph(A), sul1, sul2, tet(A), dfrA17	IncFII(pRSB107), IncFIA, IncFIB(AP001918), Col156

Five of the isolates found in environmental water samples were indistinguishable from, or closely related to, one or two isolates from patients, with ≤10 allele differences, based on cgMLST. These isolates belonged to ST38, ST394, ST949 and ST636. Further analysis of acquired resistance genes, virulence genes and plasmid replicon types within these isolates could not reveal any genetic differences in 2 isolates within ST38, 2 isolates within ST949 and the 2 isolates within ST636, while in the remaining isolates some differences were found, Table 3.

**Table 3:** Comparison of indistinguishable or closely related isolates based on cgMLST.

Isolate-ID	Source	Month of isolation	MLST	Serotype	Acquired resistance genes	Virulence genes	Plasmid replicon types
U0613-02	Urine	June	ST38	O99:H30	CTX-M-3, CTX-M-14b, aadA1, dfrA1	gad, iss, eilA, air, iha, capU, aap, aar, aatA, aggR, agg3B, agg3C, agg3D, agg5A, ORF3, ORF4	IncFII(pRSB107), IncFIB(AP001918), IncI1
SN0613-39	Svartån River	June	ST38	O99:H30	CTX-M-3, CTX-M-14b, aadA1, dfrA1	gad, iss, eilA, air, iha, capU, aap, aar, aatA, aggR, agg3B, agg3C, agg3D, agg5A, ORF3, ORF4	IncFII(pRSB107), IncFIB(AP001918), IncI1
U0713-04	Urine	July	ST38	O99:H30	CTX-M-14b, aadA1, dfrA1	gad, iss, eilA, air, iha, capU, aap, aar, aatA, aggR, agg3B, agg3C, agg3D, agg5A, ORF3, ORF4	IncFII(pRSB107), IncFIB(AP001918)
U0613-01	Urine	June	ST394	O17/O77:H18	CTX-M-15, TEM-1B, strA, strB, mph(A), sul1, sul2, tet(B), dfrA5	lpfA, eilA, air, capU, gad	IncFII(pHN7A8), IncFIB(AP001918), IncI1, Col(BS512)
EW0613-05	Effluent water from WWTP	June	ST394	O17/O77:H18	CTX-M-15, TEM-1B, strA, strB, mph(A), sul1, sul2, tet(B), dfrA5	lpfA, eilA, air, capU	IncFII(pHN7A8), IncFIB(AP001819), IncI1, Col(BS512)
U0713-01	Urine	July	ST949	O8:H11	CTX-M-15, QnrS1	gad, lpfA, iss	IncFIB(AP001918)
HF1013-17	Hjälmaren Lake	October	ST949	O8:H11	CTX-M-15, QnrS1	gad, lpfA, iss	IncFIB(AP001918), IncL/M(pMU407)
HF1013-24	Hjälmaren Lake	October	ST949	O8:H11	CTX-M-15, QnrS1	gad, lpfA, iss	IncFIB(AP001918)
U0413-05	Urine	April	ST636	O45:H7	CTX-M-15, strA, strB, aadA1, sul2, dfrA1	gad, nfaE, vat	IncFII(pRSB107), IncFIA, IncFIB(AP001918), IncQ1, Col(BS512)
HF1013-03	Hjälmaren Lake	October	ST636	O45:H7	CTX-M-15, strA, strB, aadA1, sul2, dfrA1	gad, nfaE, vat	IncFII(pRSB107), IncFIA, IncFIB(AP001918), IncQ1, Col(BS512)

The number of cgMLST allele differences: 6–10 in ST38, 0 in ST394, 7–10 in ST949, 1 in ST636.

**Fig 4:**
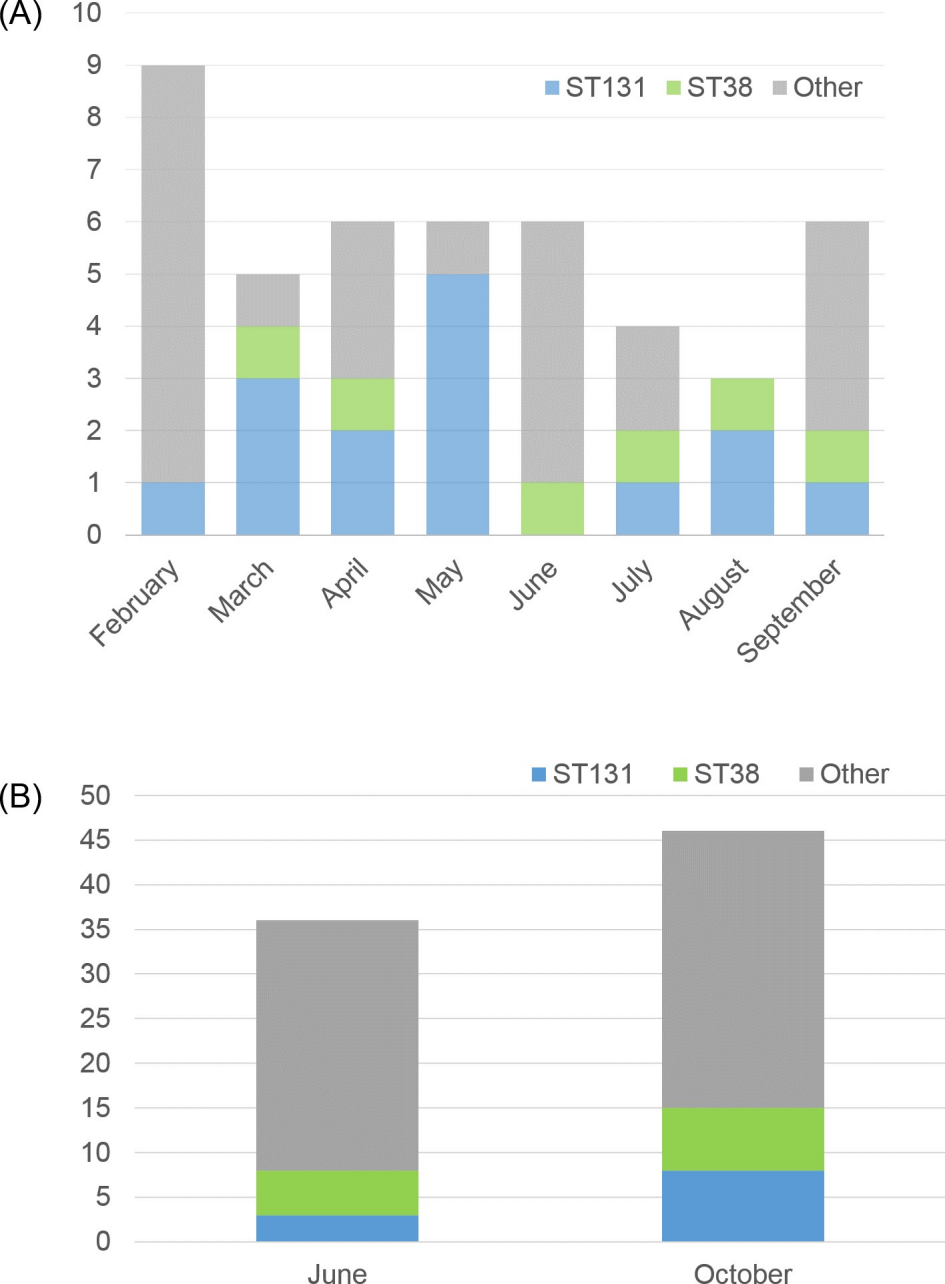
The monthly variation of ST131 and ST38. (A) The number of clinical ESBL–producing *E*. *coli* belonging to ST38, ST131, and other STs per month, 2013. (B) The number of ESBL–producing *E*. *coli* from water environments belonging to ST38, ST131, and other STs in June and October 2013.

Seasonal variations were found in the distribution of phylogenetic groups and sequence types. Among the isolates from the environment, significantly more phylogenetic group B1 isolates were found in June (*p* < 0.01), while group A isolates were more common in October (*p* < 0.01); see Table 1. Phylogenetic group B2, mainly ST131, were more common in clinical isolates collected from February to May compared to the environmental samples obtained in the beginning of June (*p* < 0.01). Conversely, phylogenetic group B1 was significantly more common in the samples from the environment (*p* < 0.01). These differences were not observed when comparing the clinical isolates found from June to September with the environmental isolates collected in the beginning of October. Fig 4 illustrates the monthly variation of STs. In February, only one clinical isolate belonged to ST131, while five out of six isolates belonged to ST131 in May. In June ST131 was not found.

## Discussion

The present study describes the molecular epidemiology of ESBL-producing *E*. *coli* isolated from humans in the clinical setting and from the aquatic environment in a country with low prevalence of antibiotic resistance. ESBL-producing *E*. *coli* lineages causing infections in humans were found to be genetically similar to those found in the aquatic environment, and isolates from different lineages were also very similar when part of the accessory genome relevant to antibiotic resistance and virulence were analyzed. The transmission of ESBL-producing *E*. *coli* in hospital environments has been extensively reported previously; however, hospitals are not the only reservoirs of antibiotic-resistant bacteria [34], as ESBL-producing *E*. *coli* have also been detected in community wastewaters [35], wild birds and animals [8, 36], and natural aquatic environments [37]. The transmission of ESBL-producing *E*. *coli* through WWTPs into the environment is considered to be a major contributor to environmental contamination [3, 17, 38]. Previous studies describing the dissemination of antibiotic-resistant bacteria focused primarily on countries with high antibiotic resistance rates.

The isolates found in the environment in the present study might have originated from various sources other than humans, such as wild birds and livestock. However, the diversity was not significantly higher in the environmental isolates compared to the clinical isolates, and the distribution of ESBL genes was also very similar, with *bla*_CTX-M-15_ being the dominating gene in both groups. The *bla*_CTX-M-15_ gene has previously been reported in the environment in other high-income countries such as Switzerland, the Netherlands, Austria, and Norway [5–7, 39].

Commensal *E*. *coli* and isolates from secondary environments (outside a host) are more likely to belong to phylogenetic groups A and B1, while extra-intestinal pathogenic strains usually are assigned to phylogenetic group B2 or D [40–42]. In the present study, isolates belonging to all these phylogenetic groups (A, B1, B2, and D) were found in both the patient samples and the environment, but as expected, group B2 was significantly more common among the clinical isolates, mainly due to a higher frequency of the pandemic lineage ST131. There was a notable variation in the monthly distribution of ST131 in the clinical samples, with a higher number during the first four months of the study period; however, the total number was relatively low. We also found that phylogenetic group B1 was the most common phylogenetic group in the environment in June, while the more genetically diverse phylogenetic group A was more common in October. A possible explanation may be that this reflects the increased international travelling during the summer months displayed by the more genetically diverse isolates.

Several clinically relevant STs were found in the environmental samples, and a few environmental and clinical isolates were found to be indistinguishable or closely related by cgMLST. The two most prevalent STs in the clinical samples, ST131 and ST38, were also the two most common types in the environment. This is in contrast to a previous national surveillance study investigating drinking water supplies in several Swedish cities, where no ST131 was detected in Örebro city and only one isolate of ST38 was found [22]. The difference might be explained by the differences in sampling strategy, since the water samples in the previous study were taken from water used as source material for drinking water and thus not directly influenced by the WWTP discharge. Both ST131 and ST38 have been reported from wastewater and environmental waters in other European countries as well [3, 5, 7, 39, 40, 43, 44]. The ST131 isolates were divided into two sub-lineages by cgMLST, corresponding to the two well-known clades: A (with serotype O16:H5 and fim*H41*) and C/H30 (with serotype O25:H4 and *fimH30*) [45, 46]. According to the cgMLST analysis, the ST131-O25:H4-*H30* isolates (lineage 2) from the environment and from patients were as closely related to each other as the isolates from different patients were. The environmental ST131-O25:H4-*H30* was, however, significantly more often associated with *bla*_CTX-M-27._ Further analysis of acquired antibiotic resistance genes, virulence genes and plasmid content, revealed that there were fewer differences between isolates carrying *bla*_CTX-M-27_ than between isolates carrying *bla*_CTX-M-15_, which could indicate that this lineage might have been introduced in this region more recently. ST131 was also the predominant ST among clinical isolates in a similar study performed in Thailand [23]. However, the isolates from the environment were in that study found to be more genetically diverse, and different *bla*_CTX-M_ dominated in the clinical collection and the environment, while the same *bla*_CTX-M_ dominated in both groups in the present study. These differences might be explained by the relatively high presence of samples collected in wastewater from farms in the Thai study. This is further supported by a recent study from the United Kingdom, where ESBL-producing *E*. *coli* in livestock were genetically distinct from isolates causing serious infection in humans [47], and no evidence of clonal spread of *E*. *coli* from farm animals to humans has been found in Sweden [48].

The results presented here are in line with previous studies suggesting that a large part of ESBL-producing *E*. *coli* present in environmental water in urban areas, such as Örebro city, probably originates from humans rather than other sources. They may consist of both commensal strains from healthy carriers in the community and pathogenic strains that reach the WWTP through the municipal wastewater system and are released into the environment. Clinical samples from healthy carriers of ESBL-producing *E*. *coli* were not included in this study, which might have affected the phylogenetic distribution. Swedish community carriers of ESBL-producing *E*. *coli* display a higher prevalence of isolates belonging to phylogroups A and B1 [15], which indicates that some of these isolates found in the environment might have a human origin. Wild birds are another possible source of contamination of the aquatic environment [36]. In this study, no notable differences were found when comparing the isolates from Oset bird sanctuary to the other environmental isolates or the clinical isolates. The samples were taken from ponds that would most likely have been highly contaminated with bacteria from bird droppings. This adds to previous data where gulls in Sweden were shown to carry ESBL-producing *E*. *coli* and ESBL genes that were genetically similar to those found in humans [49, 50].

Presence of ESBL-producing *E*. *coli* in environmental water in low-endemic settings has been described previously [7]. A recent study in Sweden has shown that 1%–4% of the total amount of *E*. *coli* found in surface water might be producing ESBL or transferrable AmpC beta-lactamases [22]. It was not within the scope of the present study to determine the prevalence of ESBL-producing *E*. *coli* in the environment. However, as the bacteria were found in every water sample from all sampling sites in the environment, it confirms that ESBL-producing *E*. *coli* now are common in the aquatic environment even in low-endemic countries such as Sweden, despite having good water purification systems and a highly restricted antibiotic usage policy. Although the samples were collected during 2013, this does not undermine the results showing that ESBL-producing *E*. *coli* from humans seems to be major contributors to environmental contamination. Carbapenemase genes or acquired genes encoding resistance to colistin were not found in the present study. However, *Klebsiella oxytoca* carrying *bla*_IMP-29_ and *bla*_VIM-1_ were recently isolated from the same WWTP and the downstream river Svartån in Örebro city [51]. The *bla*_VIM-1_ carrying isolates from these environments were genetically similar to clinical isolates from the local hospital. In a more recent study, even more carbapenemase genes were detected in Gram-negative bacteria in Svartån River and Hjälmaren Lake [52]. The levels of antibiotic resistant bacteria and resistance genes in environments receiving treated wastewater have been shown to be largely explained by fecal pollution rather than selection in the environment [38]. Taking all these findings together, there is a need to assess the capacity of WWTPs to reduce the dissemination of antibiotic-resistant bacteria into the environment.

## Conclusions

The present study confirms that ESBL-producing *E*. *coli* are common in the aquatic environment even in low-endemic countries, such as Sweden. Furthermore, the study suggests that ESBL-producing *E*. *coli* lineages are released into the environment through treated wastewater, and that lineages that are genetically similar to those causing infections in humans are present in the aquatic environment in Örebro city. This study adds to the increasing amount of evidence suggesting that additional treatment of wastewater is warranted to reduce the release of antibiotic-resistant bacteria into the environment.
